# Public demand and optimization of smart government service platform from the perspective of service life cycle based on the Kano model: A Chinese case study

**DOI:** 10.1371/journal.pone.0319707

**Published:** 2025-04-23

**Authors:** Yang Zhu, Yifan Peng, Lingling Xin, Xiao Zhang

**Affiliations:** 1 School of Economics and Management, Beijing University of Posts and Telecommunications, Beijing, China; 2 China Internet Network Information Center, Beijing, China; Department of Business Technology, Al-Ahliyya Amman University, Amman, JORDAN

## Abstract

**Background:**

Smart government is an effective way to promote government innovation and public services. However, in China, there is a mismatch between the development of smart government service platform and the demands of the public. Therefore, this study aims to clarify the categories and priorities of public demands in smart government services from service life cycle perspective.

**Methods:**

Based on the service life cycle perspective, 29 public demands in government services were summarized. A self-designed questionnaire was designed and distributed to the public using the “Jingtong” smart government service platform in Beijing. Based on 648 valid questionnaires collected, the Kano model was used to analyze the category attributes of demands, and the traditional classification and other two coefficient analysis methods were used to prioritize the demands.

**Results:**

The analysis of the collected questionnaires shows that personal privacy should be prioritized as it is the prerequisite for the widespread use of smart government services. Then, one-dimensional quality and attractive quality should be satisfied such as decision-making and intelligent scheduling demands, as they are central to the capability leap of smart government services. Additionally, must-be quality should be satisfied such as information sharing demand, as they are foundation of smart government services. Lastly, the quick response demand should be transformed into other demand types.

**Conclusions:**

This study analyzes public demands at various stages from the perspective of the service life cycle, based on the prioritization of demands, proposes targeted optimization strategies for smart government service platform in the guide, interaction, processing, and evaluation periods. This provides important references for the overall construction of smart government.

## Introduction

Smart government is an advanced product of the development of e-government at a certain stage [1]. It is a new service model that applies emerging technologies such as mobile internet, big data, cloud computing, blockchain, and artificial intelligence to the entire process of government services [2]. Smart government is an effective way to promote government innovation and public service under new circumstances [3,4]. It can effectively improve administrative efficiency, reduce operational costs, enhance transparency and accountability, and improve the quality of public services [5]. Currently, countries worldwide are vigorously developing smart government.

According to the UN E-Government Survey 2022, high EGDI (E-Government Development Index) scoring countries have increased from 69.36% to 73.38% over the past two years [6]. The United States ranks first in the Americas in terms of e-government index, focusing primarily on data openness, digital services, and smart city projects. Federal agencies like the United States Digital Service (USDS) and the 18F team promote the accessibility of public data through initiatives like Data.gov, supporting innovation and government transparency, and providing a better user experience [7,8]. In Singapore, the government is promoting digital transformation through the “Smart Nation 2025” plan, which includes smart city infrastructure, e-government services, and an artificial intelligence (AI) governance framework [9,10]. Japan ranks high among Asian countries in e-government index, primarily advancing government service digitization through the Digital Agency to improve the efficiency and convenience of public services [11]. Additionally, Japan emphasizes the application of artificial intelligence and big data technology in government decision-making to promote scientific and transparent policy-making [12]. Although China’s EGDI score lags behind that of developed countries, it is actively advancing smart government construction. By 2021, China had established a nationwide integrated government service platform, connecting all 31 provinces and cities, with real-name users exceeding 400 million. The online acceptance of provincial administrative licensing matters and the proportion of “only once” visits reached 82.13%, and the average commitment time for more than half of the national administrative licensing matters was reduced by over 40% [13]. By 2024, the national government service platform has accumulated over 890 million registered users, with over 10.7 billion identity authentication and verification services and over 10.8 billion electronic certificate sharing services provided by various regions and departments, and over 54 billion platform data sharing services have been facilitated [14]. The central government’s portal and its new media platforms cover more than 7.68 billion users in over 200 countries and regions. Meanwhile, in China, the “14th Five-Year Plan for Promoting National Government Informatization” points out that by 2025, the construction of smart government informatization will generally enter a new stage of government characterized by data empowerment, collaborative governance, smart decision-making, and high-quality services [15]. Currently, some provinces in China have achieved significant results in the construction of smart government platforms, such as “Zheliban” of Zhejiang, “Shanghai International Services”, and “Ehuiban” of Wuhan.

However, at the current stage, there is still a contradiction between the development of smart government service platforms and the alignment with public demands [16]. The construction of smart government service platforms in various countries generally follows a top-down, government-centered approach. This often leads to an inability to accurately grasp the public demands for smart government services, resulting in a mismatch between supply and demand [17]. To advance the construction of high-quality smart government service platforms, it is essential to approach it from the demand side, clarifying the public pain points and difficulties in each service process of smart government [18].

Therefore, this paper selects Beijing’s smart government service platform as the research object, focusing on four main issues:

1. How to construct a public demand analysis framework from the demand side?
2. Based on the constructed analysis framework, how to scientifically identify public demands for smart government service platforms?
3. Based on survey results, how to categorize and prioritize public demands?
4. Based on the analysis results, how to propose optimization measures for the smart government service platform?

## Literature review and analytical framework

### Literature review

At present, China has transitioned from an era of “government administrative management informatization” focused on e-government to an era of “leveraging big data to enhance the modernization of national governance” through digital government [19]. In 2012, the National Development and Reform Commission issued the “12th Five-Year Plan for the Construction of National E-Government Informatization Projects”, promoting initiatives such as “information benefiting the people” and “smart cities” nationwide. With the development of the Internet, especially mobile Internet, the communication gap between entities has shortened, improving information interaction and processing efficiency. Public demand for smart government services has genuinely extended from the physical realm into cyberspace. In 2021, the General Office of the State Council issued the “Guidelines for the Construction of Mobile Platforms for the National Integrated Government Service Platform”, emphasizing the need to address the public’s difficulties in handling affairs. It aimed to break the “last mile” of livelihood projects through services like “one-stop online processing” and “cross-region processing”. The goal at this stage is to provide a comprehensive and high-quality experience in handling matters, hoping that online and offline government service platforms can offer more personalized and humanized services [20].

The ultimate goal of building a smart government service platform to meet public demands is public satisfaction. It should be guided by public demand, meaning that the public should no longer be passive recipients of government services but should actively participate in the design and construction of these services alongside government departments [21]. Only by thoroughly identifying public demands and conducting targeted construction can the effort yield twice the results with half the effort.

However, demands are inherently the subjective perceptions of the objectively existing public, and different research perspectives can lead to varying analyses and measurements of these demands. Relevant studies have analyzed citizens’ demands for government services across various fields, such as education, healthcare, and finance [22,23]. These studies also emphasize that the government should assess and address public demands more openly, fairly, and transparently. Moreover, in constructing information systems, the government should ensure the security and privacy of personal sensitive data [24]. Other research identifies and analyzes demands from the perspectives of public behavior, personalization, and experience. For example, some studies explore factors influencing the quality of government electronic information services from the perspective of user information behavior [25], construct government information service models based on user personalized demands [26], and investigate intelligent search in government services from the user experience perspective [27]. However, these studies discuss the demands for government services that arise during user information behavior, focusing only on specific aspects of government services such as browsing, interaction, and querying. These studies are fragmented and lack a comprehensive examination of the entire process of government services.

Firstly, in terms of research methods for identifying demand, related studies either apply statistical methods to summarize and classify large-scale survey data or use mature interdisciplinary demand analysis theories to identify and analyze public demands [28,29]. Some scholars have used the ERG theory to categorize public e-government service needs into functional, relational, and growth needs and have constructed a theoretical analysis framework for public service demand research within the context of consumer decision-making and system success theories [30,31]. Some use information system success model and the technology acceptance model to examine user attitudes and satisfaction with E‐government system [32]. Others have applied the KANO model to classify public demand for online government information disclosure services into must-be qualities (M), One-dimensional qualities (O), Attractive qualities (A), and Indifferent qualities (I) [33]. Additionally, some scholars have employed lifecycle theory to study the demand identification issues of both citizen and corporate users [34] or used unsupervised statistical classification methods to analyze the demands of one million public service applications [35].

Secondly, in terms of research subjects, most existing research tends to focus on the demands arising from users’ use of online apps or government platforms. For example, some studies explore the functionality completeness of online government apps or analyze the demands and satisfaction related to government transparency on government websites [36,37], or identify barriers, successes in the digital government [38]. However, e-government should combine both online and offline components and cannot be separated.

Thirdly, most existing research targets e-government platforms, with insufficient examination of the specific characteristics of smart government service platforms. For instance, some research focuses on optimizing development approaches and governance solutions [39], or focus on personal information security and privacy [40], or investigating utilization of open government data [41]. Some research analyze the practical development logic of smart government platforms [42], explore new administrative approval models [43], and investigate comprehensive governance solutions for smart government services [44]. However, there is a lack of systematic analysis of the demands for smart government service platforms.

Building on existing research, this paper will start from the perspective of service life cycle, considering both online and offline scenarios to identify public demands within the smart government service platform, ensuring that the identification of demands is scientific, comprehensive, and systematic.

## Analytical framework

### Dimension 1: Life cycle of smart government service platform application

Smart government services aim to provide a more comfortable experience and higher satisfaction for the public. User experience includes not only feelings during processing period but also learning costs before processing and feedback after processing. To comprehensively identify public demands for smart government service platforms, this study draws on the life cycle to construct an analytical framework from a full-process of government services. The life cycle often regarded as a “Cradle-to-Grave” process and widely used in many fields such as economy, politics, and management [45].

**Fig 1:**
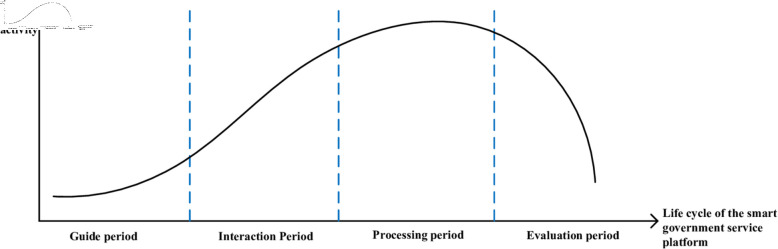
Application life cycle of smart government service platforms.

For example, the Customer Relationship Life Cycle typically refers to the entire process from establishing a business relationship with a customer to completely terminating the relationship, generally divided into four stages: inspection period, formation period, stable period, and degradation period [46]. Based on the user activity level when handling government service matters, the life cycle of smart government service platform applications is divided into four periods: information query stage before handling business (guide period), consultation and communication stage before business pre-processing (interaction period), material submission stage during business processing (processing period), and evaluation feedback stage after business completion (evaluation period), forming a “Guide Period—Interaction Period—Processing Period—Evaluation Period” life cycle, as shown in Fig 1.

### Dimension 2: Scenarios of smart government service platforms application

The application scenarios of smart government service platforms are divided into online smart government services and offline smart government services. Online smart government services mainly refer to “the public accessing government service platforms through internet methods such as mobile applications and government official websites, without geographical restrictions, and handling related government matters anytime and anywhere [47]”. Offline smart government services mainly refer to “the public arriving at government service agencies at specific times, communicating face-to-face with staff, and handling related matters [48]”.

Offline smart government services are an extension of online smart government services, with both scenarios revolving around smart government service platforms and complementing each other. In the application of smart government service platforms, online smart government services are the main focus. With the evolution of digital and smart government services, the majority of government services can be handled online, and the public prefers simple, efficient, and convenient online smart government services. Offline smart government services complement online smart government services, addressing technical blind spots, special matters, and services for special groups, providing assistance to those unfamiliar with online operations.

### Constructing the framework: Analysis framework of public demands for smartgovernment service platforms

**Fig 2:**
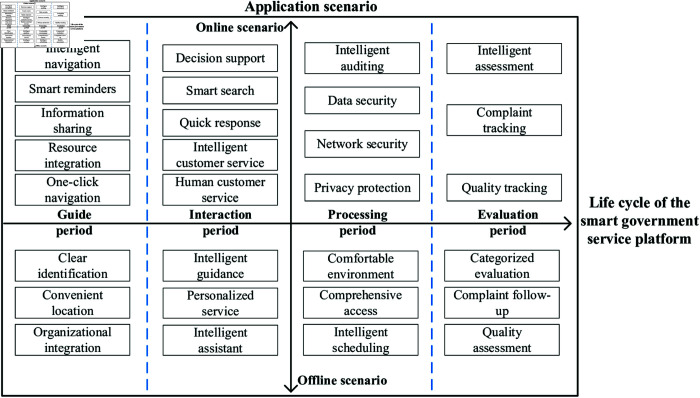
The public demand model of smart government service.

From the perspective of the service life cycle, this paper divides the life cycle of a smart government service platform into four stages: the Guide Period, the Interaction Period, the Processing Period, and the Evaluation Period. Additionally, the application scenarios are categorized into two types: online and offline (as shown in Fig 2).

During the guide period, finding the entrance to handle services quickly is public main demands. Online smart government service platforms meet the public demands for quick positioning to the processing interface through shared cloud resources, AI navigation, etc.; offline smart government services reduce the public running time through organizational integration. Online and offline smart government services complement each other to ensure accurate service entrance positioning.

During the interaction period, clearing blinding spots and obstacles before handling services is public main demands. Online smart government service platforms quickly solve personalized public issues through Artificial Intelligence Generated Content (AIGC), natural language processing (NLP), etc.; offline smart government services solve public difficult problems by enhancing staff quality. Online and offline smart government services organically combine to ensure full resolution of service difficulties.

During the processing period, ensuring the service quality is public main demands. Online smart government service platforms enhance platform security and data security through blockchain, hierarchical protection, etc., and efficiently handle processing matters through optical character recognition (OCR); offline smart government services optimize algorithms to reduce waiting time through IoT, real-time sensing of the hall status. Online and offline smart government services collaborate to ensure efficient processing of service matters.

During the evaluation period, providing objective and fair evaluation of completed matters is public main demands. Online smart government service platforms ensure objective and fair public evaluation through intelligent evaluation; offline smart government services ensure real-time tracking and processing of complaints through multi-level feedback mechanisms. Online and offline smart government services complement each other to ensure comprehensive feedback on service effects.

### Identification of public demand for smart government service platforms

Smart government is an advanced product of e-government, a specialized form of e-government services. Public demand for smart government services is a refined derivative of e-government service demand, with characteristics such as intelligence, precision, personalization, digitalization, cloud resources, AI, privacy protection, reliable transmission, and information transparency [49].

At the early stage of the research, 24 public demands were identified through literature review. Later, five experts in the fields of e-government, information management, and digital government were invited to review, modify, and add to these demands, (as shown in Table 1) resulting in the final list of 29 public demands for the smart government service platform (as shown in Table 2).

**Table 1:** Profile of expert consultation.

Expert consultation list	Title	Research field
Expert 1	Professor	E-Government and cyber space governance
Expert 2	Senior engineer	Artificial intelligence, data mining
Expert 3	Researcher	Public policy research and urban governance
Expert 4	Associate professor	5G communication, cloud computing
Expert 5	Associate researcher	Digital government

**Table 2:** Identification results of public demands for smart government service platforms.

Life cycle	Application scenario	Public demand	Demand explanation	Source
Guide period	Online	Intelligent navigation	Ensure the browsing interface has clear navigation for business projects, handling departments, and operation methods through reasonable UI design.	Reference [50]
		Smart reminders	The browsing interface includes features such as policy update notifications, appointment progress reminders, and important event alerts.	Expert consultation
		Information sharing	Government service information is shared across different departments, platforms, and systems through the establishment of standard interfaces.	Reference [51]
		Resource integration	Utilize cloud computing and other technologies to ensure comprehensive information integration and detailed classification, eliminating the need for self-querying or organizing related information.	Reference [52]
		One-click navigation	Provide intranet links for relevant departments to handle external services.	Expert consultation
	Offline	Clear identification	Utilize technologies such as IoT and 5G to update electronic tags promptly.	Expert consultation
		Convenient location	Equip a sufficient number of self-service devices and deploy them near service counters.	Reference [53]
		Organizational integration	Cluster deployment of self-service devices for different services.	Reference [54]
Interaction period	Online	Decision support	Utilize big data analytics and other technologies to assist users in filling out forms and analyzing service results.	Expert consultation
		Smart search	Utilize big data analytics and other technologies to accurately identify users’ direct search demands and potential search demands.	Reference [55]
		Quick response	Utilize cloud computing, 5G, and other technologies to ensure responsive web pages and stable site performance.	Expert consultation
		Intelligent customer service	Utilize AI, NLP, and other technologies to enable intelligent customer service to accurately understand inquiries and promptly provide effective solutions.	Expert consultation
		Human customer service	Capable of quickly transferring to human agents for personalized inquiries, providing excellent customer service with a strong professional demeanor, and offering detailed guidance.	Reference [44]
	Offline	Intelligent guidance	Utilize IoT and other technologies to assist officials in swiftly reaching service counters.	Reference [56]
		Personalized service	Establish special service counters for specific groups, such as priority windows for military personnel, disabled individuals, etc.	Expert consultation
		Intelligent assistant	Utilize speech recognition, semantic models, and other technologies to enhance the interactive experience of intelligent assistant robots.	Expert consultation
Processing period	Online	Intelligent auditing	Utilize OCR and other technologies for intelligent verification of submitted documents to facilitate efficient approval processes.	Expert consultation
		Data security	Utilize blockchain and other technologies to reconstruct database systems, enhancing the confidentiality, integrity, and security of backend data.	Expert consultation
		Network security	Utilize tiered protection and secure sockets layer (SSL) technologies to enhance platform network security, ensuring stable operation and resilience against hacking attempts.	Reference [57]
		Privacy protection	Utilize differential privacy, homomorphic encryption, and other algorithms to protect sensitive personal information from being leaked during use.	Expert consultation
		Comfortable environment	Utilize IoT and other technologies to create comfortable waiting areas, ensuring appropriate temperature and humidity, and providing supporting facilities such as service windows and reception areas, while maintaining cleanliness in public areas.	Reference [58]
		Comprehensive access	Integration and functional upgrades across desktop, lobby, mobile, and self-service terminals.	Expert consultation
		Intelligent scheduling	Utilize IoT and other technologies to sense factors such as crowd flow, service speed, and waiting times, ensuring efficient personnel distribution and smooth processing during transactions.	Expert consultation
Evaluation period	Online	Intelligent assessment	Capable of scoring each stage of business transactions and utilizing big data technology to analyze efficiency and quality of completion.	Reference [59]
		Complaint tracking	Utilize 5G and other technologies to track in real-time whether submitted suggestions have been adopted and to route complaints to relevant departments, with staff monitoring progress.	Expert consultation
		Quality tracking	Capable of conducting comprehensive evaluations of satisfaction with segmented business transactions and intelligently analyzing the reasons for low satisfaction levels.	Reference [60]
	Offline	Categorized evaluation	At service windows, allowing users to provide instant satisfaction ratings for the entire service process of staff members, enabling communication for suggestions or complaints.	Expert consultation
		Complaint follow-up	Staff promptly conduct telephone follow-up surveys to inform complainants or those who provided suggestions of the results of their concerns or feedback.	Reference [61]
		Quality assessment	Utilize IoT, sensor networks, and other technological means to monitor quality indicators at each stage of the service process.	Reference [62]

## Research design

### Research approach

**Fig 3:**
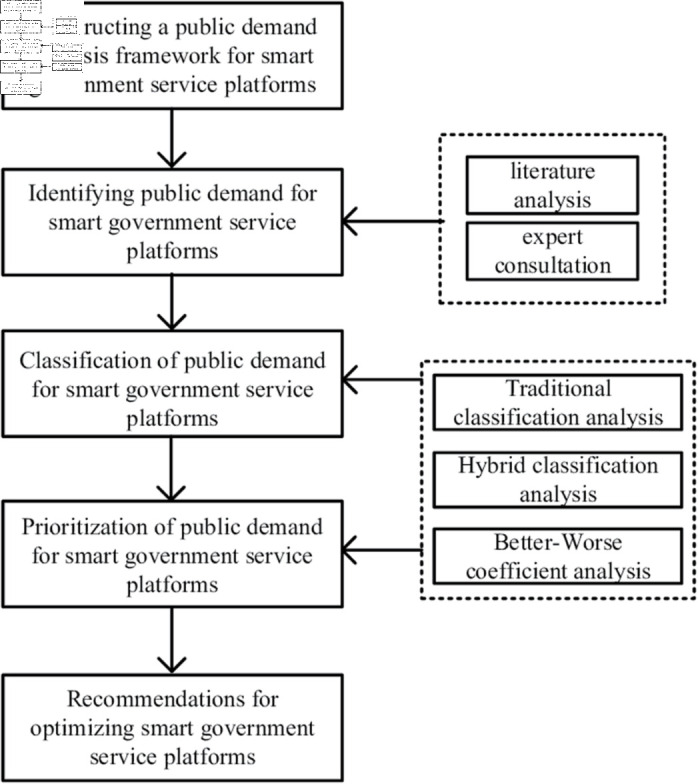
Research approach.

The research approach is shown in Fig 3.

### Method

**Fig 4:**
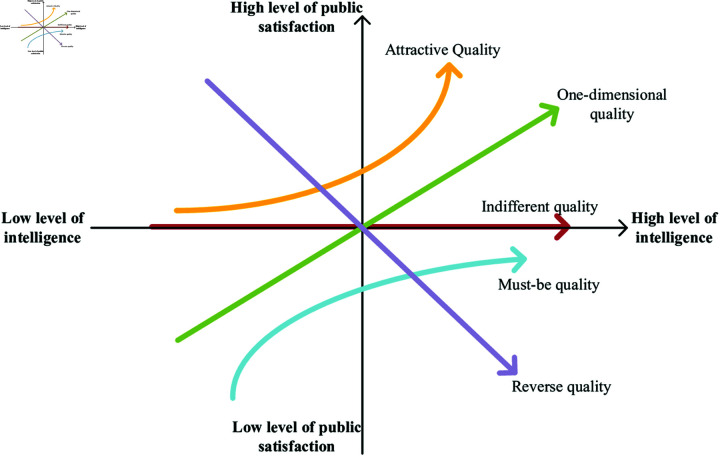
Kano model.

In this paper, the Kano model is selected as the theoretical analysis method. The Kano model is a tool for categorizing and prioritizing user demands. It was proposed by Noriaki Kano in 1984 [63], who linked user demands with satisfaction levels regarding a product or service. Kano model identifies five types of factors that influence satisfaction: Must-be qualities (M), One-dimensional qualities (O), Attractive qualities (A), Indifferent qualities (I), and Reverse qualities (R), as shown in Fig 4. Must-be qualities are attributes or functions of a platform or service that the public considers “must-have”. In this paper, it means that if this need is not met, the public will be very dissatisfied; however, even if the need is met, it does not necessarily increase public satisfaction. One-dimensional qualities are attributes or functions that the public expect the platform or service to achieve. In this paper, it means that if this need is not met, the public will be very dissatisfied; but if the need is fulfilled, the public will feel satisfied. Attractive qualities are attributes or functions of a platform or service that the public consider to be “beyond expectations”. In this paper, it means that if this need is not met, the public will not be dissatisfied; however, if this need is met, it will pleasantly surprise the public and greatly enhance their satisfaction. Indifferent qualities are attributes or functions that the public consider to be “optional”. In this paper, it means that regardless of whether this need is met, the public will not care, nor will it affect their satisfaction or dissatisfaction. Reverse qualities are attributes or functions that users or the public consider the platform or service “should not provide”. In this paper, it means that this need is negatively correlated with public satisfaction, and providing it may decrease public satisfaction instead.

Two reasons for selecting this model. First, compared to other demand analysis theories such as the ERG model, the Kano model considers user demands from both an objective perspective (i.e., the impact of the completeness of a service on user demands) and a subjective perspective (i.e., user satisfaction). Second, the Kano model is widely used in e-government demand research [64–66], as it not only highlights the different levels of public demands but also emphasizes high-level public demands. For these reasons, using the Kano model as the theoretical research method can thoroughly clarify and identify the pain points and difficult demands of the public throughout the service life cycle of government services. In addition, the research has got ethics approval application form of Beijing University of Posts and Telecommunications.

## Research data

### Questionnaire design

The questionnaire was designed strictly according to the Kano model methodology. Initially, 50 questionnaires were distributed in a small-scale pilot, and based on the results and feedback from the pre-survey, the questionnaire was revised and improved. The final questionnaire is divided into three parts: the first part contains the title and instructions, explaining the purpose of the survey, the content of the questionnaire, and the use of the data collected; the second part focuses on the respondents’ basic information, including gender, age, Physical condition, degree, occupation, and their purpose of smart government services; the third part addresses the main content of the questionnaire, where questions are based on the public demands identified in the four periods of the smart government services life cycle (as shown in Table 3). A total of 29 questions are set: 8 for the guide period, 8 for the interaction period, 7 for the processing period, and 6 for the evaluation period. The functional questions assume the service or function is provided, while the dysfunctional questions assume the service or function is not provided. Each question uses a five-point Likert scale for respondents to choose their answers: “Dislike”, “Live with”, “Neutral”, “Must-be”, and “Like ” (as shown in Table 4) [65,67].

**Table 3:** Kano questionnaire example.

The browsing interface of the online government platform has clear navigation for business items, departments, and operational methods	Dislike	Live with	Neutral	Must-be	Like
Functional question: If the service or function is provided, how do you feel?					*✓*
Dysfunctional question: If the service or function is not provided, how do you feel?		*✓*			

**Table 4:** Classification of the Kano requirement evaluation.

Question type	Functional: If (the service) satisfied (requirement x), how would you feel?					
Dysfunctional: If (the service) did not satisfy (requirement x), how would you feel?	Answer	Like	Must-be	Neutral	Live with	Dislike
	Like	Q	R	R	R	R
	Must-be	A	I	I	I	R
	Neutral	A	I	I	I	R
	Live with	A	I	I	I	R
	Dislike	O	M	M	M	Q

“O” represents one-dimensional, “M” represents must-be, “A” represents attractive, “I” represents indifferent, “R” represents reverse, and “Q” represents questionable.

### Questionnaire distribution and collection

“Jingtong” was chosen as the research object for the following reasons: (1) Launched in October 2023, “Jingtong” integrates the services of “Beijing Tong” and “Beijing Health Treasure”, serving as the mobile gateway for urban public services in the construction of a smart city. (2) “Jingtong” covers a wide range of application areas, offering one-stop services from healthcare to cultural and tourism activities. It updates and iterates its services rapidly, having already added functions such as mobile payment for medical insurance and a big data platform for rural revitalization. (3) “Jingtong” actively utilizes cutting-edge technologies such as AI, big data, and cloud computing. It embodies the digitalization, cloud resource integration, and AI features that a smart government service platform should have, such as storing personal information on Beijing’s government cloud.

From December 1, 2023 to March 31, 2024, electronic questionnaires were distributed to the public using Beijing’s “Jingtong” smart government service platform. A random sampling method was adopted, with a total of 695 questionnaires distributed. By including a question asking, “Have you used the ‘Jingtong’ platform?” respondents were screened, and suspicious or invalid questionnaires were excluded. A total of 648 valid questionnaires were collected, resulting in an effective response rate of 93.23%.

**Table 5:** Statistics of sample characteristics.

Essential information	Option	Sample book	Proportion (%)
Age	18–30 years old	90	13.89
	31–45 years old	237	36.57
	46–60 years old	189	29.17
	61–70 years old	86	13.27
	Over 70 years old	46	7.1
Physical status	Health	622	95.99
	Physical disability	26	4.01
Record of formal schooling	Junior high school and below	108	16.67
	Senior middle school	154	23.76
	Undergraduate and junior college	222	34.26
	Master Degree candidate	108	16.67
	Doctoral candidate	56	8.64
Occupation	Full-time student	58	8.95
	Government/public institutions	70	10.8
	State-owned enterprises	131	20.22
	Private enterprise	134	20.68
	Individual operation	114	17.59
	Other professions	141	21.76
The purpose of using the “Jingtong”	Browse and query	117	18.06
	Consult, q & A	174	26.85
	Issue processing	229	35.34
	Complaint evaluation	128	19.75

### Sample characteristics

Respondents had balanced gender ratios, reasonable distributions of age, physical status, education, occupation, and purposes for using “Jingtong”, as shown in Table 5).

Samples with similar characteristics exhibit convergent demands for smart government service platforms [68]. Therefore, after conducting scientific sampling, a small sample survey still holds representativeness.

### Questionnaire quality

The questionnaire’s quality allows for analysis. Cronbach’s alpha coefficients for functional and dysfunctional questions were 0.789 and 0.806, respectively, indicating good consistency and stability. KMO values for functional and dysfunctional questions were 0.867 and 0.873, indicating the questions effectively reflect public demand.

**Table 6:** Classification of public demands for smart government service platforms.

Life cycle	Application scenario	Public demand	Demand category						Kano type	TS	CS	Mixed type	SI	DSI	B-W type
			A	O	M	I	R	Q							
Guide period	Online	Intelligent navigation	145	158	147	149	24	25	O	0.694	0.014	H(O+I)	0.506	− 0 . 509	O
		Smart reminders	158	166	127	125	38	34	O	0.696	0.012	H(O+A)	0.563	− 0 . 509	O
		Information sharing	83	86	228	194	35	22	M	0.613	0.052	H(M+I)	0.286	− 0 . 531	M
		Resource integration	196	80	82	198	59	33	I	0.552	0.003	I	0.496	− 0 . 291	A
		One-click navigation	50	89	267	183	38	21	M	0.627	0.13	M	0.236	− 0 . 604	M
	Offline	Clear identification	89	110	223	162	39	25	M	0.651	0.094	M	0.341	− 0 . 57	M
		Convenient location	267	29	42	219	62	29	A	0.522	0.074	A	0.531	− 0 . 127	A
		Organizational integration	187	190	83	142	24	22	O	0.71	0.005	H(O+A)	0.626	− 0 . 453	O
Interaction period	Online	Decision support	121	209	146	118	30	24	O	0.735	0.097	O	0.556	− 0 . 598	O
		Smart search	145	193	124	137	25	24	O	0.713	0.074	O	0.564	− 0 . 529	O
		Quick response	99	56	135	265	59	34	I	0.448	0.201	I	0.279	− 0 . 344	I
		Intelligent customer service	234	102	63	170	43	36	A	0.616	0.099	A	0.591	− 0 . 29	A
		Human customer service	140	129	154	160	38	27	I	0.653	0.009	H(I+M)	0.461	− 0 . 485	M
	Offline	Intelligent guidance	231	131	77	159	34	16	A	0.677	0.111	A	0.605	− 0 . 348	A
		Personalized service	91	70	216	184	52	35	M	0.582	0.049	M	0.287	− 0 . 51	M
		Intelligent assistant	256	88	86	173	29	16	A	0.664	0.128	A	0.57	− 0 . 289	A
Processing period	Online	Intelligent auditing	138	207	140	130	21	12	O	0.748	0.103	O	0.561	− 0 . 564	O
		Data security	62	87	253	174	41	31	M	0.62	0.122	M	0.259	− 0 . 59	M
		Network security	54	168	267	118	24	17	M	0.755	0.153	M	0.366	− 0 . 717	M
		Privacy protection	159	253	97	73	36	30	O	0.785	0.145	O	0.708	− 0 . 601	O
	Offline	Comfortable environment	77	80	233	208	30	20	M	0.602	0.039	H(M+I)	0.263	− 0 . 523	M
		Comprehensive access	206	110	72	147	67	46	A	0.599	0.091	A	0.591	− 0 . 34	A
		Intelligent scheduling	277	66	63	199	27	16	A	0.627	0.12	A	0.567	− 0 . 213	A
Evaluation period	Online	Intelligent assessment	225	138	68	174	18	25	A	0.665	0.079	A	0.6	− 0 . 34	A
		Complaint tracking	238	65	71	226	38	10	A	0.577	0.019	A	0.505	− 0 . 227	A
		Quality tracking	158	216	117	110	26	21	O	0.758	0.09	O	0.622	− 0 . 554	O
	Offline	Categorized evaluation	235	55	64	221	51	22	A	0.546	0.022	A	0.504	− 0 . 207	A
		Complaint follow-up	237	62	62	184	79	24	A	0.557	0.082	A	0.549	− 0 . 228	A
		Quality assessment	147	154	129	138	44	36	O	0.664	0.011	H(O+A)	0.53	− 0 . 498	O

## Result

### Classification of public demands for smart government service platforms

In the Kano model classification analysis, the option with the highest occurrence in the questionnaire is considered as the attribute of the demand. Based on this, the collected questionnaire data is subjected to the classification analysis as shown in the Table 6. According to these criteria, 10 attractive qualities (A), 9 one-dimensional qualities (O), 7 must-be qualities (M), 3 indifferent qualities (I), and 0 reverse quality (R) were identified for the smart government service platform.

### Attribute correction of public demand for smart government service platformbased on hybrid class analysis

After the preliminary classification using traditional methods, situations with similar frequencies exist, making it difficult to clarify dominant demands. Lee et al. proposed the use of hybrid class analysis to determine the developmental trends of demand elements’ category attributes [69]. The TS (Total Strength) index reflects whether respondents would be satisfied with the demand element (as shown in Eq 1), while CS (Category Strength) reflects the degree of acceptance of the classification results(as shown in Eq 2).

$$\begin{array}{ccc} TS=\frac{M+O+A}{M+O+A+I+R+Q} & & \end{array}$$ (1)

$$\begin{array}{ccc} CS=\frac{\left[\max(M,O,A,I,R,Q)-second\max(M,O,A,I,R,Q)\right]}{M+O+I+R+Q} & & \end{array}$$ (2)

When the total strength TS is equal or greater than 60% and the category strength CS is equal or smaller than 6%, it is classified as a hybrid category (H), based on the top two demand categories by frequency ranking. The attribute correction results are as follows:

1. H(M+I) includes the demands for comfortable environment, information sharing, and human customer service. This indicates the necessity of incorporating these functions into the smart government service platform. Continuous optimization of these functions is required to facilitate the transition to stable must-be quality.
2. H(O+I) includes the demand for intelligent navigation. This indicates that such demands are attractive to the public and require improvement in supply quality to prevent them from becoming indifferent qualities.
3. H(O+A) includes the demands for intelligent reminders, quality assessment, and organizational integration. This suggests that the public is sensitive to the high-quality supply of such services. They may be excited by the platform’s user-friendly operation, timely progress notifications, and abundant self-service facilities. Therefore, enhancing the public’s experience is necessary.

The types of demand elements will change over time [70], following the trend of I→A→O→M.

### Classification presentation of public demand for smart government service platform based on Better-Worse coefficient analysis

The hybrid analysis lacks consideration of the distribution of other categories with slightly lower frequencies. Therefore, Berger C. et al. proposed the Satisfaction Index (Better-Worse coefficient) to analyze the impact of demand on public satisfaction [71]. The Better coefficient (satisfaction coefficient, SI) reflects the increase in public satisfaction when a demand is met. This value is usually greater than 0, and the closer to 1 indicates the higher influence of this demand element on public satisfaction, as shown in Eq 3. The Worse coefficient (dissatisfaction coefficient, DSI) reflects the decrease in public satisfaction when a demand is not met. This value is usually less than 0, and the closer to –1 indicates the higher influence of this demand element on public dissatisfaction, as shown in Eq 4.

$$\begin{array}{ccc} Better(SI)=\frac{O+A}{M+O+A+I} & & \end{array}$$ (3)

$$\begin{array}{ccc} Worse(DSI)=-\frac{M+O}{M+O+A+I} & & \end{array}$$ (4)

**Fig 5:**
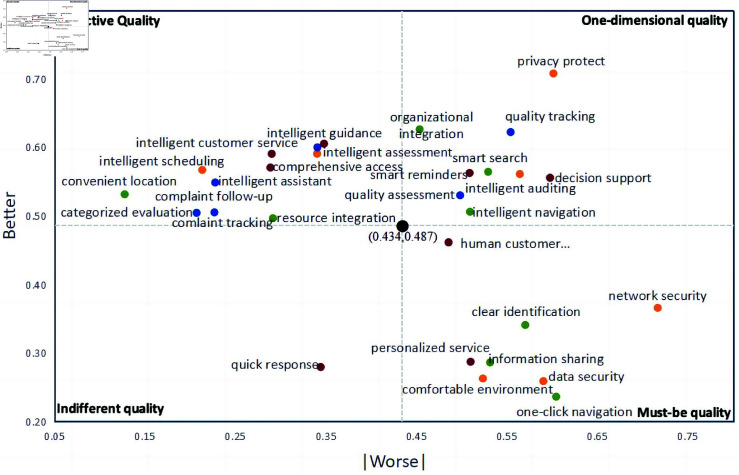
Better-Worse coefficient distribution chart of public demand for smart government service.

A quadrant chart is plotted with the average absolute values of the Better-Worse coefficient as the origin (0.434, 0.487) (Fig 5). The presentation of results is as follows:

1. Emphasize one-dimensional qualities of the smart government service platform. Provide sufficient supply to enhance user experience and meet user expectations.
2. Highlight attractive qualities of the smart government service platform. Improve supply as much as possible to maintain happiness and enhance user stickiness.
3. Indifferent qualities of the smart government service platform can be considered as weaknesses. Promote their transformation into attractive quality, enhance the sense of acquisition, and stimulate the generation of new demands.
4. Focus on must-be qualities of the smart government service platform as the baseline. Ensure high-quality supply to provide a sense of security and guarantee user trust.

### Summary analysis

Among the three analytical methods, 27 demands show high consistency in their classification results, ultimately categorized as follows:

1. Attractive quality include intelligent scheduling, convenient location, intelligent customer service, intelligent guidance, intelligent assistant, comprehensive access, intelligent assessment, complaint tracking, categorized evaluation, and complaint follow-up.
2. One-dimensional quality include decision support, smart search, intelligent navigation, smart reminders, intelligent auditing, organizational integration, privacy protection, quality tracking, and quality assessment.
3. Must-be quality include information sharing, clear identification, comfortable environment, personalized service, data security, network security, and one-click navigation.
4. Only the quick response requirement falls under the category of no difference.

There are two requirements with inconsistent results among the three analytical methods: human customer service and resource integration. Human customer service is categorized as no difference in the traditional classification. After hybrid analysis, it appears as both no difference and must-be quality. According to the Better-Worse coefficient analysis, it appears as a must-be quality. This indicates that the functionality of human customer service does not affect the public’s use of the smart government service platform. However, for certain groups, such as the elderly, human customer service may impact satisfaction, suggesting the need for ongoing maintenance of the platform. Resource integration is categorized as no difference in both traditional and hybrid analyses. According to the Better-Worse coefficient analysis, it appears as a attractive quality. This suggests that resource integration enhances public satisfaction with the smart government service platform.

### Priority ranking of public demands for smart government service platform

The smart government service platform represents the further evolution of e-government service platform business scenarios and technological capabilities. To maximize public satisfaction, the following basic sorting principles will be followed: first, fully enhance the supply of one-dimensional quality, second, strive to improve the supply of attractive quality, third, ensure the supply of must-be quality, and finally, promote the transformation of indifferent quality. Therefore, according to the sequence of the first quadrant, second quadrant, fourth quadrant, and third quadrant in Fig 5, the Better-Worse values are calculated respectively and sorted from largest to smallest [72]. As shown in Table 7 for the sorting results.

**Table 7:** Prioritization of public demand for smart government service.

Life cycle	Application scenario	Public demand	Demand type	Sort
Guide period	Online	Intelligent navigation	O	9
		Smart reminders	O	7
		Information sharing	M	26
		Resource integration	A	15
		One-click navigation	M	25
	Offline	Clear identification	M	23
		Convenient location	A	20
		Organizational integration	O	6
Interaction period	Online	Decision support	O	3
		Smart search	O	5
		Quick response	I	29
		Intelligent customer service	A	13
		Human customer service	M	22
	Offline	Intelligent guidance	A	10
		Personalized service	M	27
		Intelligent assistant	A	14
Processing period	Online	Intelligent auditing	O	4
		Data security	M	24
		Network security	M	21
		Privacy protection	O	1
	Offline	Comfortable environment	M	28
		Comprehensive access	A	12
		Intelligent scheduling	A	16
Evaluation period	Online	Intelligent assessment	A	11
		Complaint tracking	A	18
		Quality tracking	O	2
	Offline	Categorized evaluation	A	19
		Complaint follow-up	A	17
		Quality assessment	O	8

It is worth mentioning that the choice of the above ranking principle in this paper is based on the premise that questionnaire responses are somewhat subjective, and the analysis is relatively simple and intuitive. The Better-Worse ranking method allows for the quick completion of demand priority ranking without the need for complex methods such as AHP or TOPSIS. If further decomposition of the demand hierarchy and determination of weights are required, AHP can be chosen; if the relative advantages and disadvantages of the options need to be considered, TOPSIS can be selected.

## Discussion

The optimization approach for smart government service platforms is as follows: First, priority should be given to ensuring personal privacy security, as this is the precondition for widespread use of smart government services. Existing studies [73] have analyzed the phenomenon of collection of personal information by platforms. The results of this analysis show that the public values personal privacy security the most. As the development of intelligent government systems requires more personal information to support business processes, the focus should be on designing robust privacy protection mechanisms for the platform. Subsequently, the platform should prioritize its development according to one-dimensional qualities, attractive qualities, and must-be qualities at different periods. Finally, efforts should be made to transform indifferent qualities into other types of qualities.

### Guide period: Focus on enhancing smart push effects

The analysis results show that during the guidance period, the public’s expectations for smart reminders and intelligent navigation are particularly urgent. Existing research [74] has analyzed the public’s demand for aesthetically pleasing interfaces. However, the practical challenge is that as more government services become available online, it becomes difficult for the public to find the services they need through complicated interfaces. Government service platforms should focus on using new media technologies to enhance reminder efficiency. For example, they could implement reasonable functional partitions and hierarchical navigation in the interface design. Regarding offline government services, existing research [75] primarily emphasizes the integrated development of online and offline services. The analysis results suggest focusing on developing organizational integration of one-dimensional qualities,reducing the spatial distance and time consumption for the public to access services, and further integrating different departments and services within a unified physical area. For instance, emphasis could be placed on using technologies such as the Internet of Things and sensor networks to strengthen organizational integration capabilities, or deploying various types of self-service robots in government service halls.

### Interaction period: Emphasize improving human-computer interactionefficiency

The analysis results indicate that one-dimensional quality such as decision support and smart search, as well as intelligent customer service, are particularly urgent. However, there is limited literature analyzing these demands. As the number of online services provided by government platforms increases, so do the requirements for processing these services. The reality is that even if the public finds the service they need, complex processing requirements often leave them feeling at a loss. Optimizing these demands can help the public save time better on inquiries and improve service efficiency. Government service platforms should further utilize technologies such as AI, big data analysis, and cloud computing to enhance human-machine interaction efficiency. For example, deep reinforcement learning algorithms, continuous training of semantic large models, and deploying OpenStack cloud computing models could all be used to improve question-answering efficiency.

Offline smart government service platforms should focus on developing attractive quality for intelligent guidance and intelligent assistants. Optimizing these demands can reduce the time spent by the public in handling affairs. For offline smart government service platforms, there should be a focus on developing intelligent guidance and intelligent assistant needs, which differs from the focus of existing research [76]. At this period, it is crucial to utilize various sensors to perceive the status of the public as a group and as individuals, or to apply natural language processing technology to intelligent assistant robots to enhance human-machine interaction efficiency.

### Processing period: Focus on enhancing intelligent processing efficiency

The processing period is the most critical phase in determining the effectiveness of public service. Existing research [73] has examined the collection of personal information by platforms. This study further identifies that the public places significant emphasis on the expectations for privacy protection and intelligent auditing. In practice, it is often observed that, despite submitting application materials, the public faces prolonged manual review processes, and in some cases, their applications are returned for revisions before resubmission. Government service platforms should prioritize the application of technologies such as Optical Character Recognition (OCR) and Multi-Level Security Protection to enhance the efficiency of intelligent auditing and safeguard privacy. For instance, regular security assessments by professional institutions can ensure platform security, while the integration of convolutional neural networks and Hadoop-based big data analytics can significantly improve intelligent auditing processes.

For offline smart government service platforms, the focus should be on developing the attractive qualities for cross-regional and cross-departmental integrated service capabilities, thereby improving the efficiency of joint service delivery. This aligns with existing research findings [77]. During this period, it is essential to focus on utilizing virtualization technologies such as KVM and Docker. This ensures that government service applications can run smoothly on different operating systems such as Android, HarmonyOS, iOS, Windows, Linux, and Kirin, thereby achieving linkage and functionality upgrades across computer terminals, lobby terminals, mobile terminals, and self-service terminals.

### Evaluation period: Ensure real-time adaptive dynamic feedback

The analysis results indicate that the public demand for attractive qualities such as intelligent assessment and one-dimensional quality like quality tracking is particularly urgent. Previous research [78] has analyzed the factors influencing satisfaction with smart government services. This study expands upon those factors, suggesting that quality tracking should also be considered as a key element. Government service platforms should focus on leveraging technologies such as 5G and big data analytics to enhance evaluation and tracking capabilities.For example, the service process can be broken down into detailed stages with scoring options to ensure comprehensive coverage of public evaluation needs. Additionally, AI and big data technologies can be used to analyze the efficiency and quality of service completion, providing recommendations for improvement.

For offline platforms, there should be a focus on the development of one-dimensional quality such as quality evaluation. Existing research [79] has primarily emphasized efficiency evaluation. This study, however, further identifies the importance of the public real-time sensory experience during service processes. For instance, it is possible to utilize IoT sensors to monitor indicators such as queue length, queue time, environmental temperature, and humidity in real time. Backend algorithms can then be employed to dynamically adjust the environmental conditions, ensuring the real-time experience of the public.

## Conclusion

The innovations and theoretical contributions of this paper are as follows: First, it introduces an innovative framework for analyzing public demand on government service platforms across both online and offline scenarios from a service life cycle perspective, which has not been addressed in existing studies. The life cycle perspective ensures the completeness of process analysis, while considering both online and offline contexts ensures comprehensive scenario analysis. Future researchers can adopt this analytical framework to construct demand frameworks for more specific areas. Second, based on the current state of government service capabilities in China, the paper identifies 29 types of public demand attributes and their importance, reflecting actual public needs and providing a reference for evaluating the current level of government service capabilities in China. Finally, unlike previous studies that focus solely on either management or technology, this paper simultaneously proposes optimization strategies for smart government service platforms from both technical and managerial perspectives.

## Limitations

This paper analyzes the actual public demand using Beijing’s government service platform as the research subject, without taking into account specific factors such as varying levels of economic development across different administrative regions or the age distribution of major population groups. Future research will focus on specific regions or demographic groups. Although this study employs hybrid class analysis and the Better-Worse coefficient analysis to rank various demands, for deeper analysis, future studies could consider combining a more systematic Analytical Hierarchy Process (AHP) and the Technique for Order of Preference by Similarity to Ideal Solution (TOPSIS), which allows for the ranking of priorities among specific options.
